# The mitochondria‐targeted anti‐oxidant MitoQ protects against intervertebral disc degeneration by ameliorating mitochondrial dysfunction and redox imbalance

**DOI:** 10.1111/cpr.12779

**Published:** 2020-02-05

**Authors:** Liang Kang, Shiwei Liu, Jingchao Li, Yueyang Tian, Yuan Xue, Xiaozhi Liu

**Affiliations:** ^1^ Department of Orthopedics Tianjin Medical University General Hospital Tianjin China; ^2^ Tianjin Key Laboratory of Spine and Spinal Cord Injury Tianjin China; ^3^ Department of Orthopedics Tianjin Jinghai District Hospital Tianjin China; ^4^ Central Laboratory The Fifth Central Hospital of Tianjin Tianjin China

**Keywords:** apoptosis, intervertebral disc degeneration, mitochondrial dynamics, mitophagic flux, MitoQ, Nrf2

## Abstract

**Objective:**

Mitochondrial dysfunction, oxidative stress and nucleus pulposus (NP) cell apoptosis are important contributors to the development and pathogenesis of intervertebral disc degeneration (IDD). Here, we comprehensively evaluated the effects of mitochondrial dynamics, mitophagic flux and Nrf2 signalling on the mitochondrial quality control, ROS production and NP cell survival in in vitro and ex vivo compression models of IDD and explored the effects of the mitochondria‐targeted anti‐oxidant MitoQ and its mechanism.

**Material and methods:**

Human NP cells were exposed to mechanical compression to mimic pathological conditions.

**Results:**

Compression promoted oxidative stress, mitochondrial dysfunction and NP cell apoptosis. Mechanistically, compression disrupted the mitochondrial fission/fusion balance, inducing fatal fission. Concomitantly, PINK1/Parkin‐mediated mitophagy was activated, whereas mitophagic flux was blocked. Nrf2 anti‐oxidant pathway was insufficiently activated. These caused the damaged mitochondria accumulation and persistent oxidative damage. Moreover, MitoQ restored the mitochondrial dynamics balance, alleviated the impairment of mitophagosome‐lysosome fusion and lysosomal function and enhanced the Nrf2 activity. Consequently, damaged mitochondria were eliminated, redox balance was improved, and cell survival increased. Additionally, MitoQ alleviated IDD in an ex vivo rat compression model.

**Conclusions:**

These findings suggest that comodulation of mitochondrial dynamics, mitophagic flux and Nrf2 signalling alleviates sustained mitochondrial dysfunction and oxidative stress and represents a promising therapeutic strategy for IDD; furthermore, our results provide evidence that MitoQ might serve as an effective therapeutic agent for this disorder.

## 1 INTRODUCTION

Intervertebral disc (IVD) degeneration (IDD) is widely acknowledged to be the primary cause of low back pain (LBP)—a chronic, expensive and common musculoskeletal disorder that is prevalent in developed and developing countries.^1^ The IVD consists of three inter‐related structures: the nucleus pulposus (NP), the annulus fibrosus (AF) and the cartilaginous endplate. The centrally situated NP allows the IVD to maintain a high water content and thereby withstand mechanical impact.^2^ Excessive apoptosis of NP cells can trigger metabolic disorders within the NP extracellular matrix, resulting in the destruction of normal IVD structure and physiological function that eventually leads to IDD.^2^, ^3^ Thus, determination of the key molecular mechanisms of NP cell apoptosis would be of great significance to the management of IDD.

The maintenance of a healthy and functional mitochondrial network is critical during development as well as for physiological adaptations and responses to stress in the body.^4^, ^5^ Mitochondria are not only the main cellular source of reactive oxygen species (ROS), they are also particularly susceptible to oxidative injury.^6^ Recently, studies have verified the presence of oxidative stress and increased concentrations of oxidation products in degenerated discs.^7^, ^8^, ^9^ Additionally, oxidative stress and subsequent mitochondrial dysfunction participate in the intrinsic pathway of cellular apoptosis, which have been confirmed in NP cell death and IDD induced by various risk factors.^10^, ^11^, ^12^, ^13^, ^14^, ^15^ Moreover, mitochondrial dysfunction induced by ROS can further enhance the production of ROS, leading to a feed‐forward vicious cycle between mitochondria and ROS that causes sustained oxidative damage.^16^ Thus, in addition to direct intervention targeting oxidative stress, the role of appropriate mitochondrial quality control in NP cell survival under pathological conditions is also worth studying.

Mitochondria are highly dynamic organelles that continuously undergo fission and fusion, known as mitochondrial dynamics.^17^ The subtle equilibrium of mitochondrial dynamics is conducive to the maintenance of a healthy pool of mitochondria.^18^ Destruction of this equilibrium is implicated in various human diseases including cancer, type 2 diabetes and osteoarthritis.^4^, ^19^, ^20^ In addition to mitochondrial dynamics, the timely and selective removal of damaged mitochondria through an autophagic process termed mitophagy is important for maintaining mitochondrial quality.^5^ Impairment of mitophagic flux results in the accumulation of dysfunctional mitochondria and ROS associated with various diseases.^21^, ^22^ It has been shown that mitophagy and mitochondrial dynamics are inter‐related yet distinct processes. During mitochondrial fission, the damaged daughter mitochondria are first separated then targeted by the lysosome for elimination, preventing damaged mitochondria from being incorporated back into the active and healthy mitochondrial pool via fusion.^4^

The occurrence of oxidative stress caused by ROS overproduction is inseparable from anti‐oxidant system defects. Nuclear factor E2‐related factor 2 (Nrf2) is a key redox‐sensitive transcription factor that regulates the anti‐oxidant defence system by activating the expression of a number of cytoprotective genes in response to oxidative stress.^23^ In addition, there is evidence that Nrf2 modulates mitochondrial function and metabolism.^24^ Therefore, the present study investigated the maintenance of mitochondrial homeostasis via the simultaneous regulation of mitochondrial dynamics, mitophagy and Nrf2 signalling using a pharmacological method to rescues mitochondrial dysfunction and NP cell apoptosis under pathological conditions.

Mitoquinone (MitoQ)—a mitochondria‐targeted anti‐oxidant—consists of co‐enzyme Q10 and a TPP cation that can easily accumulate several 100‐fold within the mitochondria, thereby making it more powerful than untargeted anti‐oxidants in preventing mitochondrial oxidative damage.^25^ In vitro and in vivo studies have demonstrated that MitoQ is protective against many oxidative damage‐related diseases.^26^, ^27^, ^28^, ^29^, ^30^, ^31^, ^32^, ^33^ In addition, daily oral doses of 40 or 80 mg MitoQ have been shown to protect against liver damage in hepatitis C patients^34^ and can be safely administered to Parkinson's disease patients for up to 1 year.^35^ There is evidence that the protective effect of MitoQ is partially mediated by the modulation of mitochondrial dynamics, mitophagy and Nrf2 signalling.^33^, ^36^ These potential therapeutic benefits are encouraging and warrant further investigation of MitoQ as an intervention to prevent the development of IDD.

As a load‐absorbing structure of the spine, the IVD is subjected to various magnitudes of mechanical compression throughout daily life.^37^, ^38^, ^39^ Inappropriate or excessive compression applied to IVDs is an important contributing factor in causing IDD.^10^, ^40^, ^41^, ^42^, ^43^, ^44^ Therefore, the in vitro and ex vivo models of IDD induced by compression are more in line with the pathogenesis of IDD. Studies have reported that ROS overproduction, mitochondrial dysfunction and apoptosis were induced by compression at a magnitude of 1.0 MPa in NP cells, all of which were closely related to IDD.^10^, ^11^, ^45^, ^46^ Thus, we for the first time systematically investigated the changes in mitochondrial dynamics, mitophagy and Nrf2 signalling in human NP cells under 1.0 MPa compression. In addition, we studied the effect of MitoQ on human NP cells, as well as the roles of mitochondrial dynamics, mitophagy and Nrf2 signalling in the action of MitoQ. An ex vivo compression model of rat tail disc degeneration was used to confirm the results of the in vitro experiments. Our aim was to provide novel insights for the development of effective therapeutic strategies to inhibit IDD progression.

## 2 MATERIAL AND METHODS

### 2.1 Tissue specimens and cell culture

This study was approved by the Ethics Committee of Tianjin Medical University General Hospital, and written informed consent was obtained from each donor. Nucleus pulposus specimens were collected from 10 IDD patients (6 males and 4 females, 41‐69 years of age) suffering from cervical spondylotic myelopathy who received anterior cervical discectomy and fusion at Tianjin Medical University General Hospital. Nucleus pulposus cell isolation and culture were carried out as previously described.^47^ During passaging, no significant changes in morphology were observed between primary (passage 0) and later‐passage (passage 2) cells. Therefore, we used second‐passage cells cultured in a monolayer for experiments.

### 2.2 Establishment of ex vivo organ culture of IVD

IVDs were collected from Sprague Dawley rats (3 months old) with the ethical approval of the Animal Care and Use Committee of Tianjin Medical University. The intact caudal discs with complete endplates were isolated and cultured in DMEM containing 15% foetal bovine serum (Gibco) and 1% penicillin/streptomycin (Invitrogen) as previously described.^48^

### 2.3 Compression treatment

IVD organs or NP cells were exposed to continuous high pressure in a previously described compression apparatus.^10^, ^48^ In brief, the tissues or cells were placed in cell culture plates on the bottom of the compression apparatus, which contained a small amount of distilled water to maintain the moisture level. The compression apparatus was placed in an incubator at 37°C. A mixture of 0.5% CO_2_ and 99.5% compressed air was pumped into the apparatus until the pressure reached 1.0 MPa. The control group samples were incubated at 37°C without any compression under the same culture conditions.

### 2.4 Cell viability assay

According to the manufacturer's instructions, the cytotoxic effect of compression on human NP cells was assessed using a cell counting kit (CCK‐8; Dojindo). Briefly, the cells were seeded in 96‐well plates and treated with MitoQ or compression. Subsequently, the cells were incubated with 10 μL CCK‐8 solution at 37°C for 2 hours. The absorbance at 450 nm was measured using a spectrophotometer (BioTek).

Stock solutions of MitoQ (Focus Biomolecules) were prepared by dissolving it in dimethyl sulfoxide (DMSO) (Sigma‐Aldrich). The stock solutions were subsequently diluted with culture medium. The final concentrations of DMSO in the culture medium were ≤0.1% (v/v). Appropriate controls containing DMSO were included in all experiments.

### 2.5 Western blotting

After cell treatments, total, cytoplasmic and mitochondrial proteins from NP cells were extracted using commercial kits (Beyotime) according to the manufacturer's instructions. Protein concentrations were determined with a BCA Protein Assay Kit (Beyotime). Equal amounts of protein from each sample were separated using SDS‐PAGE and transferred to PVDF membranes. The membranes were blocked with 7.5% non‐fat milk for 1 hour and then incubated at 4°C overnight with the following primary antibodies: cytochrome c (ab133504, Abcam), cleaved caspase‐3 (#14220, Cell Signaling Technology), Drp1 (ab56788, Abcam), Mff (#84580, Cell Signaling Technology), Fis1 (ab71498, Abcam), Mid49 (bs‐12633R, Bioss), Mid51 (bs‐12634R, Bioss), Mfn1 (#14739, Cell Signaling Technology), Mfn2 (#9482, Cell Signaling Technology), Opa1 (ab42364, Abcam), Parkin (ab77924, Abcam), PINK1 (ab23707, Abcam), p62 (ab56416, Abcam), LC3 (#12741, Cell Signaling Technology), Beclin‐1 (#3495, Cell Signaling Technology), Nrf2 (ab137550, Abcam), Keap1 (10503‐2‐AP, Proteintech), SOD‐2 (24127‐1‐AP, Proteintech), HO‐1 (10701‐1‐AP, Proteintech) and NQO‐1 (11451‐1‐AP, Proteintech). GAPDH (#5174, Cell Signaling Technology), COX IV (11242‐1‐AP, Proteintech) and Lamin B (12987‐1‐AP, Proteintech) were used as internal controls. The membranes were subsequently incubated with the respective secondary antibodies (Abcam) at room temperature for 1 hour. Protein bands were visualized by the enhanced chemiluminescence method (Amersham Biosciences) according to the manufacturer's instructions. Band intensities were quantified using ImageJ software (NIH).

### 2.6 Short interfering (si)RNA transfection

SiRNAs against PINK1 (siPINK1) (5′‐GCUAGUUACAAGAGAACAA‐3′), Parkin (siParkin) (5′‐GAAUACAUUCCCUACCUCA‐3′), Nrf2 (siNrf2) (5′‐GAGAAAGAAUUGCCUGUAA‐3′), and HO‐1 (siHO‐1) (5′‐CAGUUGCUGUAGGGCUUUA‐3′) and scrambled siRNA (si‐Control) (5′‐UUCUCCGAACGUGUCACGU‐3′) were designed and synthesized by RiboBio. Nucleus pulposus cells were transfected for 48 hours with 100 nmol/L of each siRNA using Lipofectamine® 2000 (Invitrogen) according to the manufacturer's instructions. The cells were then used for experiments.

### 2.7 Flow cytometry

Apoptosis levels, ΔΨm changes, mPTP opening, cellular ROS production and mitochondrial ROS production in human NP cells from each treatment group were assessed using an Annexin V‐APC/7‐AAD Apoptosis Detection Kit (Yeasen), JC‐1 Assay Kit (Beyotime), mPTP Assay Kit (Yeasen), DCFH‐DA (Beyotime) and MitoSOX Red (Invitrogen), respectively, as previously described.^15^ After labelling, samples were examined using a FACSCalibur flow cytometer (BD Biosciences).

### 2.8 Measurement of malondialdehyde (MDA) levels

MDA levels were measured using assay kits for MDA (Beyotime) as per manufacturer's instructions.

### 2.9 Immunofluorescence

Immunofluorescence staining of human NP cells was conducted as described previously.^49^ The cells were incubated with primary antibodies against Tom20 (mitochondrial antibody) (ab186734, Abcam), Drp1 (ab56788, Abcam), Parkin (ab77924, Abcam), LC3 (#12741, Cell Signaling Technology), LAMP1 (lysosomal antibody) (ab25630, Abcam) and Nrf2 (ab137550, Abcam) at 4°C overnight and then with appropriate secondary antibodies at room temperature for 1 hour. Nuclei were stained with DAPI (ZSGB‐BIO). Fluorescence images were acquired using a laser‐scanning confocal microscope (FV1000; Olympus) or confocal laser‐scanning microscope (LSM800; Zeiss).

### 2.10 mRFP‐GFP‐LC3 assay

Human NP cells were infected with mRFP‐GFP‐LC3 adenoviral vectors (HanBio Technology) to evaluate the effects of compression and MitoQ on mitophagic flux. The principle of the assay is based on differences in pH stability between the green and red fluorescent proteins. The mRFP and GFP puncta in each treatment group were detected using a laser‐scanning confocal microscope (FV1000; Olympus).

### 2.11 Measurement of cathepsin B (CTSB) activity, cathepsin D (CTSD) activity and lysosomal pH changes

CTSB and CTSD activities were measured using specific fluorometric assay kits for the two enzymes (BioVision) as described previously.^50^ Changes in lysosomal pH were determined using LysoSensor Green DND‐189 (Yeasen) as described previously^51^; this reagent exhibits a pH‐dependent increase in fluorescence intensity in acidic environments.

### 2.12 Assessments of IVD ex vivo compression model

The rat disc tissues from each group were harvested. The specimens were fixed in formaldehyde, decalcified, dehydrated, embedded in paraffin and sectioned at a thickness of 4 μm. The sections were stained with haematoxylin and eosin (HE) and safranin O‐fast green (SO), and the histological grades of the specimens were determined to quantify damage based on a previously described method.^52^ For TUNEL staining, the sections were handled using a TUNEL Apoptosis Assay Kit (Beyotime) and samples were imaged with a fluorescence microscope (Olympus IX71). Immunohistochemistry was performed as described previously.^53^ For immunohistochemical analysis, sections were incubated with primary antibodies against cleaved caspase‐3 (#9664, Cell Signaling Technology), Drp1 (ab56788, Abcam), Mfn2 (#9482, Cell Signaling Technology), Parkin (ab77924, Abcam), LC3 (#3868, Cell Signaling Technology), P62 (ab56416, Abcam) and Nrf2 (ab137550, Abcam) at 4°C overnight and then with the appropriate secondary antibodies. Moreover, cleaved caspase‐3, Drp1, Mfn2, PINK1, Parkin, P62, Nrf2 and LC3 expression levels in the disc tissues were detected by Western blotting according to the procedure described in Section 2.5. Lastly, the content of H_2_O_2_ in the disc tissues was assessed using a Hydrogen Peroxide Assay Kit (Nanjing Jiancheng Bioengineering Institute) according to the manufacturer's instructions.

### 2.13 Statistical analysis

Data are presented as the mean ± standard deviation (SD) of at least three independent experiments and were analysed using SPSS version 18.0 software (SPSS Inc, Chicago, IL, USA). Differences between groups were evaluated using Student's *t* test or one‐way analysis of variance (ANOVA) followed by Tukey's test. *P* < .05 was considered statistically significant.

## 3 RESULTS

### 3.1 Effects of MitoQ on compression‐induced cytotoxicity in human NP cells

**Figure 1:**
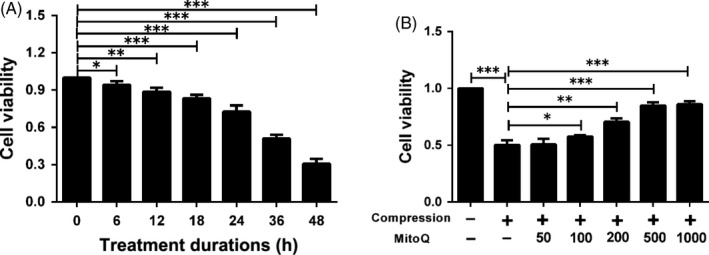
MitoQ protects human NP cells against compression‐induced cytotoxicity. (A) CCK‐8 assay was used to determine the cytotoxic effects of compression on human NP cells by exposing the cells to 1.0 MPa compression for indicated time points (6, 12, 18, 24, 36 and 48 h). (B) The viability of the human NP cells pre‐treated with different concentrations (nmol/L) of MitoQ for 2 h and then exposed to 1.0 MPa compression for 36 h was determined by CCK‐8 assay. Data are represented as the mean ± SD. ***P < .001, **P < .01, *P < .05, n = 3

Compression was used to establish an IDD model in vitro. As shown in Figure 1A, the 1.0 MPa compression reduced the viability of human NP cells in a time‐dependent manner from 0 to 48 hours. Considering that compression treatment for 36 hours resulted in a 49% reduction in cell viability and that this time point of 36 hours has been used in several studies to induce oxidative stress, mitochondrial dysfunction and apoptosis in NP cells,^10^, ^11^, ^45^, ^46^ we used this 36 hours time point in subsequent experiments. In addition, our results showed that MitoQ provided substantial protection against this compression‐induced reduction in cell viability (Figure 1B). The difference in viability between the 500 nmol/L‐ and 1000 nmol/L‐treated groups was significantly smaller than that between the 200 nmol/L‐ and 500 nmol/L‐treated and 100 nmol/L‐ and 200 nmol/L‐treated groups. Therefore, MitoQ was used at doses of 200 and 500 nmol/L in the following experiments.

### 3.2 Effects of MitoQ on compression‐induced ROS accumulation, mitochondrial dysfunction and apoptosis in human NP cells

**Figure 2:**
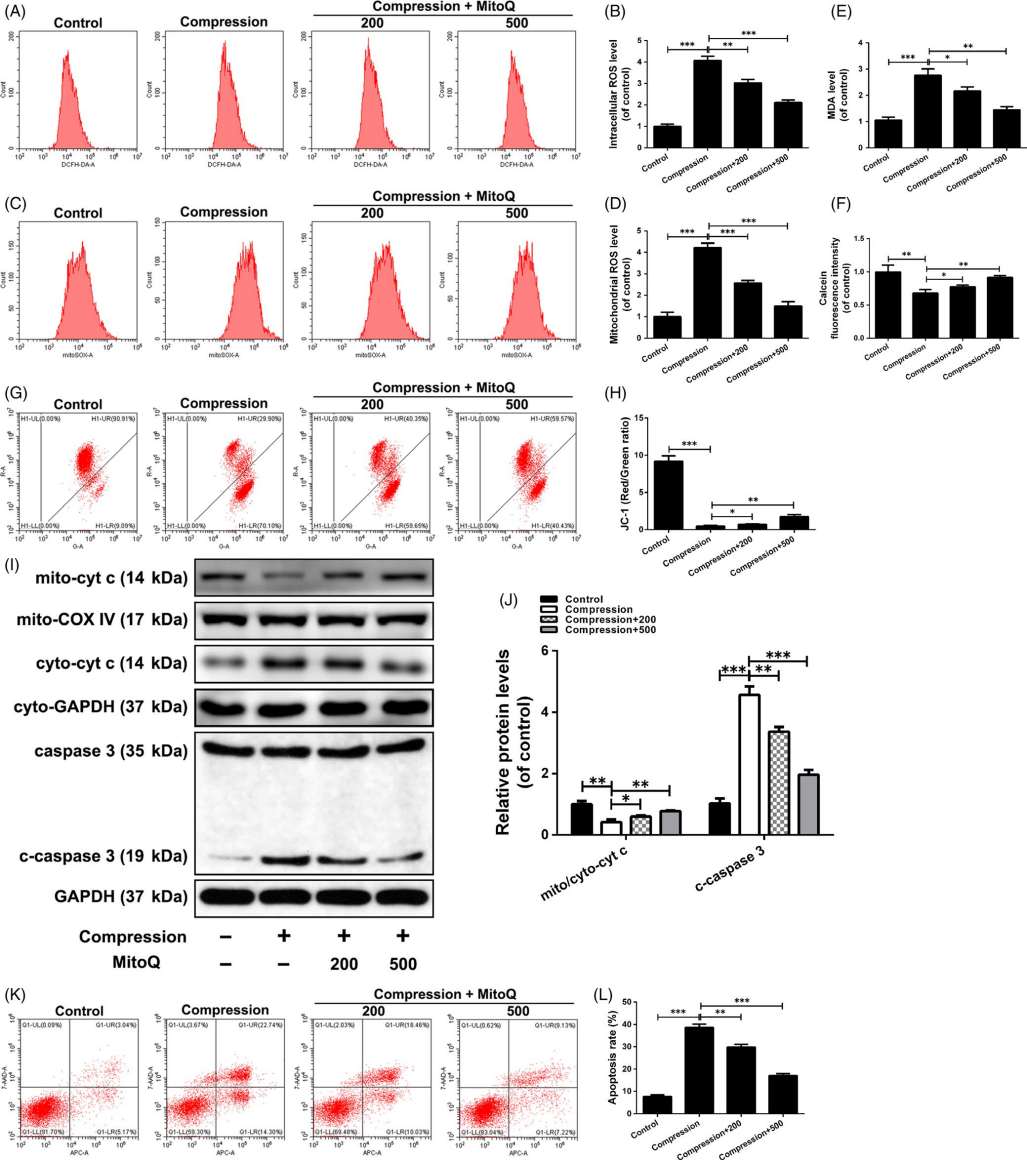
Effect of MitoQ on compression‐induced ROS accumulation, mitochondrial dysfunction and apoptosis in human NP cells. Human NP cells were pre‐treated with MitoQ (200 nmol/L, 500 nmol/L) for 2 h and then exposed to compression for 36 h. (A‐B) The intracellular ROS levels in the human NP cells were detected using the fluorescent probe DCFH‐DA and measured by flow cytometry. (C‐D) The mitochondrial ROS levels were detected using the mitochondrial ROS‐specific dye MitoSOX Red and measured by flow cytometry. (E) Intracellular MDA levels in the human NP cells. (F) Calcein fluorescence intensity in mitochondria represents opening level of mPTP, which is measured by flow cytometry. (G‐H) Mitochondrial membrane potential was detected by JC‐1 staining and measured by flow cytometry. (I‐J) The protein levels of mitochondrial cytochrome c (mito‐cyt c), cytoplasmic cytochrome c (cyto‐cyt c), caspase‐3 and cleaved caspase‐3 (c‐caspase 3) in the human NP cells were measured by Western blotting. (K‐L) Annexin V‐APC/7‐AAD staining results showing the rate of apoptosis in human NP cells. Data are represented as the mean ± SD. ***P < .001, **P < .01, *P < .05, n = 3

Next, we investigated whether MitoQ protects NP cells from compression‐induced damage. The results showed that compression treatment significantly increased intracellular and mitochondrial ROS production in human NP cells and that this was inhibited by MitoQ (Figure 2A‐D). MDA levels in human NP cells were markedly increased after exposure to compression (Figure 2E). MitoQ largely suppressed the elevation in MDA levels induced by compression (Figure 2E). Flow cytometric analysis indicated that excessive mitochondrial ROS production was accompanied by increased mPTP opening (Figure 2F) and decreased ΔΨm (Figure 2G,H) in human NP cells exposed to compression. However, this alteration was largely abolished by MitoQ (Figure 2F‐H). Finally, MitoQ alleviated the increase in human NP cell apoptosis induced by compression, as determined by Western blotting and flow cytometry (Figure 2I‐L).

### 3.3 Effects of MitoQ on compression‐induced alterations in mitochondrial dynamics in human NP cells

**Figure 3:**
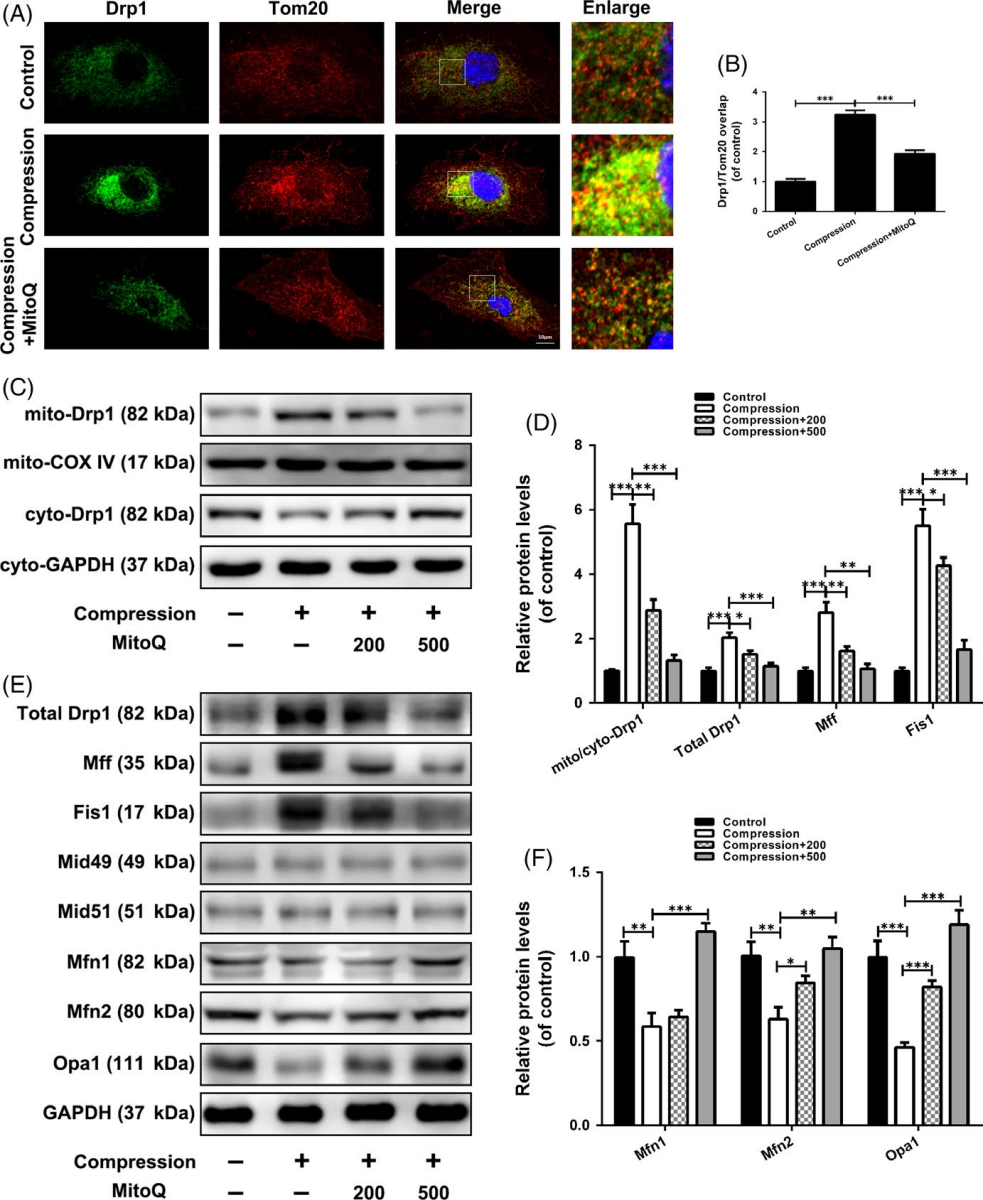
Effects of MitoQ on compression‐induced alterations in mitochondrial dynamics in human NP cells. (A‐B) Human NP cells were pre‐treated with MitoQ (500 nmol/L) for 2 h and then exposed to compression for 36 h. The colocalization of Drp1 and Tom20 was examined by confocal microscopy. Scale bar: 10 μm. (C‐D) The protein levels of mitochondrial Drp1 (mito‐Drp1) and cytoplasmic Drp1 (cyto‐Drp1) in the human NP cells were measured by Western blotting. (D‐F) The protein levels of total Drp1, Mff, Fis1, Mid49, Mid51, Mfn1, Mfn2 and Opa1 in the human NP cells were measured by Western blotting. Data are represented as the mean ± SD. ***P < .001, **P < .01, *P < .05, n = 3

Migration of Drp1 from the cytoplasm to mitochondria is a pre‐requisite for mitochondrial fission. As shown in Figure 3A,B, compression treatment enhanced the translocation of Drp1 to the mitochondria, as evidenced by the stronger colocalization of Drp1 with mitochondria in compression‐exposed cells compared to that in control cells, whereas MitoQ alleviated the compression‐induced translocation of Drp1. Next, Western blotting showed that compression treatment resulted in an increase in the mitochondrial Drp1 level and a concomitant decrease in the cytosolic level (Figure 3C,D). Total Drp1 protein expression was obviously upregulated in human NP cells stimulated by compression as compared to control cells (Figure 3D,E). MitoQ attenuated compression‐induced alterations in the total protein level and intracellular distribution of Drp1 (Figure 3C‐E). Drp1 is recruited to the surface of mitochondria to constrict them, a process that requires the assistance of its receptors Mff, Fis1, Mid49 and Mid51. As shown in Figure 3D,E, Mff and Fis1 protein levels were significantly upregulated, whereas Mid49 and Mid51 showed no significant changes in compression‐exposed cells compared to control cells. MitoQ decreased the protein levels of Mff and Fis1, but not those of Mid49 and Mid51 (Figure 3D,E).

Among the proteins responsible for mitochondrial fusion, Mfn1, Mfn2 and Opa1 protein levels were reduced after compression treatment; however, this reduction was ameliorated by MitoQ intervention (Figure 3E,F). These results indicated that MitoQ can rescue the imbalance between mitochondrial fission and fusion induced by compression in human NP cells.

### 3.4 Effects of MitoQ‐maintained mitochondrial dynamics balance on compression‐induced damage in human NP cells

**Figure 4:**
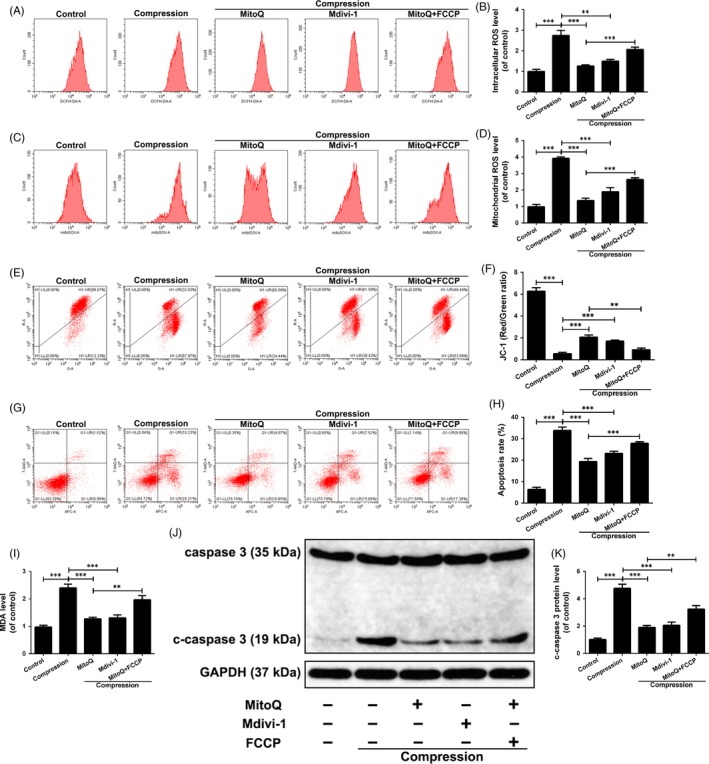
Effects of MitoQ‐maintained mitochondrial dynamics balance on compression‐induced damage in human NP cells. Human NP cells were pre‐treated with MitoQ (500 nmol/L) for 2 h or Mdivi‐1 (20 μmol/L) for 4 h prior to the compression treatment for 36 h. Before the administration of MitoQ and compression, human NP cells were pre‐treated with 5 μmol/L of FCCP for 30 min. (A‐B) The intracellular ROS levels in the human NP cells were detected using the DCFH‐DA and measured by flow cytometry. (C‐D) The mitochondrial ROS levels were detected using the MitoSOX Red and measured by flow cytometry. (E‐F) Mitochondrial membrane potential was detected by JC‐1 staining and measured by flow cytometry. (G‐H) Annexin V‐APC/7‐AAD staining results showing the rate of apoptosis in human NP cells. (I) Intracellular MDA levels in the human NP cells. (J‐K) The protein levels of caspase 3 and c‐caspase 3 in the human NP cells were measured by Western blotting. Data are represented as the mean ± SD. ***P < .001, **P < .01, *P < .05, n = 3

We next investigated whether these beneficial effects of MitoQ are attributed to rectifying the imbalance of mitochondrial fusion and fission in human NP cells treated with compression. In the compression treatment group, cells were treated with Mdivi‐1, a blocker of mitochondrial fission. In the compression and MitoQ cotreatment group, cells were treated with FCCP, an agonist of mitochondrial fission. Like MitoQ intervention, Mdivi‐1 significantly suppressed the elevations in ROS generation, mitochondrial impairment and apoptosis induced by compression treatment (Figure 4A‐K). The observed beneficial effects of MitoQ intervention were partially inhibited by FCCP (Figure 4A‐K). Collectively, these results suggested that the maintenance of the balance between mitochondrial fission and fusion is critical for the maintenance of mitochondrial homeostasis and NP cell survival under compression.

### 3.5 Effects of MitoQ on PINK1/Parkin‐mediated damaged mitochondrial clearance in human NP cells exposed to compression

**Figure 5:**
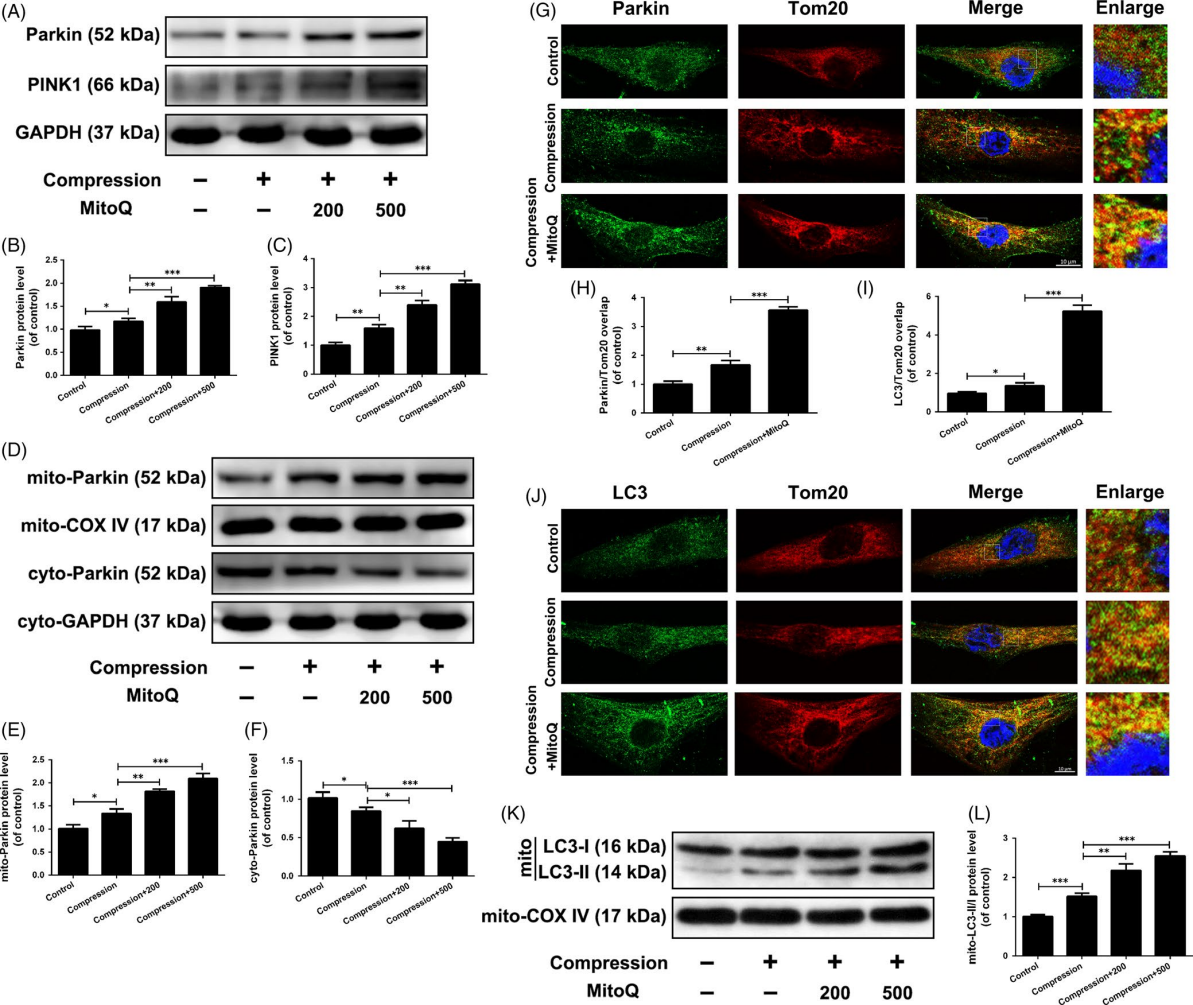
Effects of MitoQ on PINK1/Parkin pathway‐mediated mitophagy in human NP cells exposed to compression. (A‐F) Human NP cells were pre‐treated with MitoQ (200 nmol/L, 500 nmol/L) for 2 h and then exposed to compression for 36 h. (A‐C) The protein levels of Parkin and PINK1 in the human NP cells were measured by Western blotting. (D‐F) The protein levels of mito‐Parkin and cyto‐Parkin in the human NP cells were measured by Western blotting. (G‐J) Human NP cells were pre‐treated with MitoQ (500 nmol/L) for 2 h and then exposed to compression for 36 h. (G‐H) The colocalization of Parkin and Tom20 was examined by confocal microscopy. Scale bar: 10 μm. (I‐J) The colocalization of LC3 and Tom20 was examined by confocal microscopy. Scale bar: 10 μm. (K, L) The protein levels of mitochondrial LC3 in the human NP cells were measured by Western blotting. Data are represented as the mean ± SD. ***P < .001, **P < .01, *P < .05, n = 3

We first investigated whether the PINK1/Parkin signalling pathway was activated in human NP cells exposed to compression. Western blot results showed that the total protein expression of PINK1 and Parkin increased after compression treatment (Figure 5A‐C), whereas the cytosolic Parkin level decreased and the mitochondrial Parkin level increased (Figure 5D‐F). In addition, co‐immunostaining of Parkin and Tom20 in human NP cells revealed an elevated colocalization of Parkin and Tom20 in compression‐exposed cells compared to control cells (Figure 5G,H). Next, we assessed the effect of compression in the next step of mitophagy, the engulfment of mitochondria by autophagosomes. The immunofluorescence results showed that compression treatment promoted colocalization of LC3 and Tom20 (Figure 5I,J). Moreover, Western blot analysis indicated that the protein level of mitochondrial LC3‐II was increased in compression‐exposed cells compared with control cells (Figure 5K,L). These results suggested that compression activates PINK1/Parkin pathway‐mediated mitophagy.

**Figure 6:**
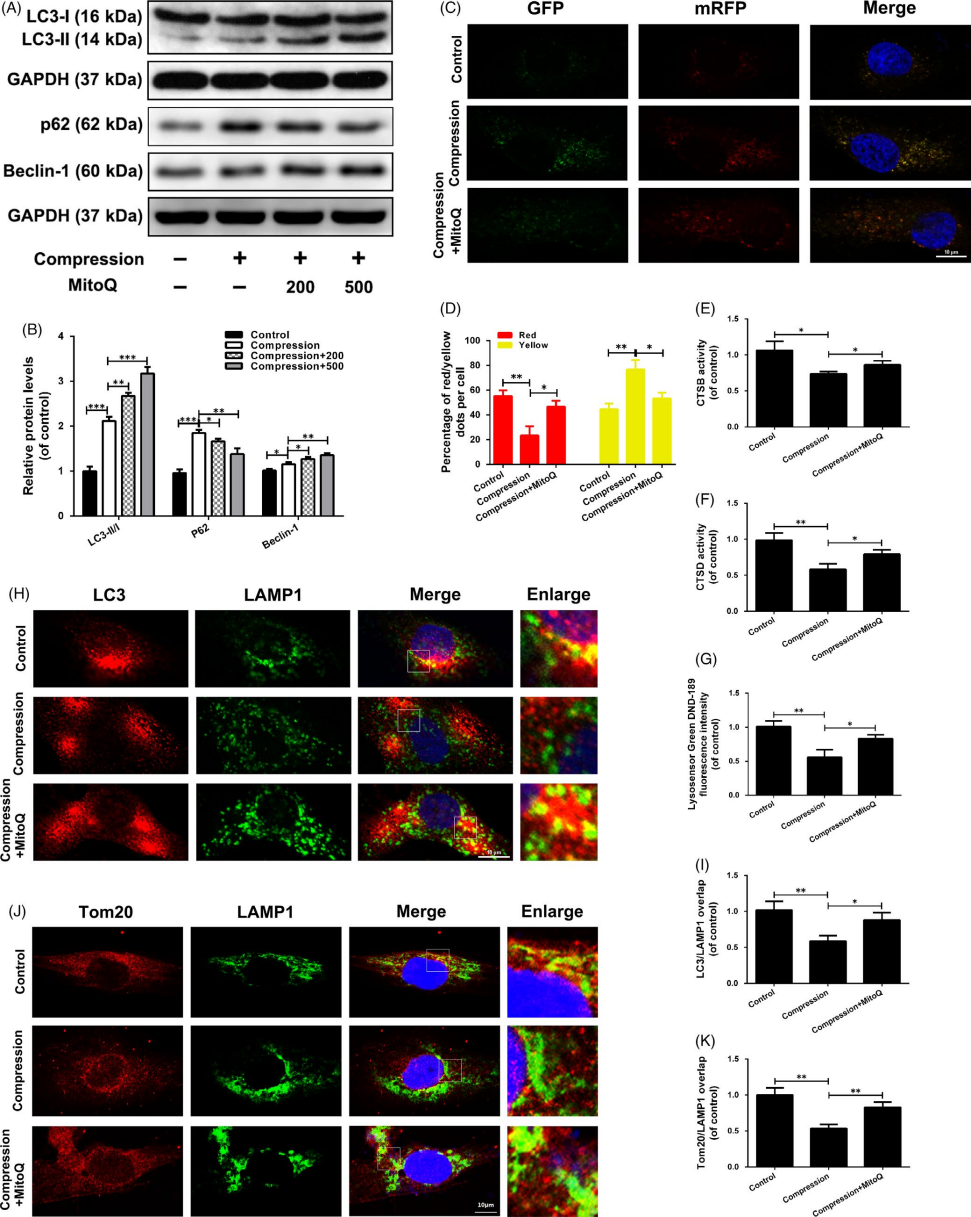
MitoQ repairs defective mitophagic flux in human NP cells exposed to compression. (A‐B) The protein levels of LC3, p62 and Beclin‐1 in the human NP cells were measured by Western blotting. (C‐D) Representative images of the human NP cells expressing mRFP‐GFP‐LC3 were obtained by confocal microscopy. Scale bar: 10 μm. (E‐F) The activity of CTSB and CTSD in the human NP cells. (G) Lysosomal pH of human NP cells was determined by LysoSensor Green DND‐189 staining. (H‐I) The colocalization of LC3 and LAMP1 was examined by confocal microscopy. Scale bar: 10 μm. (J‐K) The colocalization of Tom20 and LAMP1 was examined by confocal microscopy. Scale bar: 10 μm. Data are represented as the mean ± SD. ***P < .001, **P < .01, *P < .05, n = 3

Dysfunctional mitochondria are delivered to lysosomes for degradation following their engulfment in autophagosomes, a process that requires undamaged mitophagic flux. Western blot results indicated that compression treatment markedly increased the p62 protein level, but did not decrease the LC3‐II/I ratio and Beclin‐1 protein level (Figure 6A,B). This finding was confirmed by transfection of a tandem mRFP‐GFP‐LC3 construct into human NP cells. As compared with the control group, the compression group showed an increase in yellow but not red puncta (Figure 6C,D). These data suggested that the mitophagic flux was impaired in compression‐exposed human NP cells.

Next, we explored the mechanism underlying mitophagic flux blockade by compression. The activities of CTSB and CTSD—key proteases that degrade trapped cargo in lysosomes—were measured to evaluate lysosomal degradation ability in the treated human NP cells. As shown in Figure 6E,F, compression decreased the activity levels of both enzymes. Low lysosomal pH is required for the maturation and activation of most lysosomal enzymes; thus, we measured the lysosomal pH by LysoSensor Green DND‐189 staining. As shown in Figure 6G, compression treatment induced an obvious decrease in fluorescence intensity. Further, we investigated mitophagosome‐lysosome fusion based on LC3‐LAMP1 colocalization. Compression led to a significant decrease in LC3‐LAMP1 colocalization when compared to the control groups (Figure 6H,I). The colocalization of Tom20 and LAMP1 was decreased in compression‐exposed cells compared to control cells (Figure 6J,K), suggesting that dysfunctional mitochondria engulfed in mitophagosomes would not be able to enter lysosomes in compression‐exposed human NP cells. These data suggested that compression impairs lysosomal function and mitophagosome‐lysosome fusion.

Notably, MitoQ further promoted PINK1 and Parkin protein expression (Figure 5A‐C), Parkin recruitment to the mitochondria (Figure 5D‐H) and mitophagosome formation (Figure 5I‐L) in compression‐exposed human NP cells. MitoQ enhanced p62 protein degradation, the LC3‐II/I ratio, Beclin‐1 protein expression and GFP quenching of the tandem mRFP‐GFP‐LC3 construct (Figure 6A‐D). MitoQ restored lysosomal protease activity and acidity and promoted the colocalization of LAMP1 with LC3 and Tom20 (Figure 6E‐K). Collectively, these findings suggested that MitoQ promotes PINK1/Parkin‐mediated mitophagy and repairs defective mitophagy flux in human NP cells exposed to compression.

### 3.6 Role of mitophagic flux in the beneficial effects of MitoQ on compression‐exposed human NP cells

**Figure 7:**
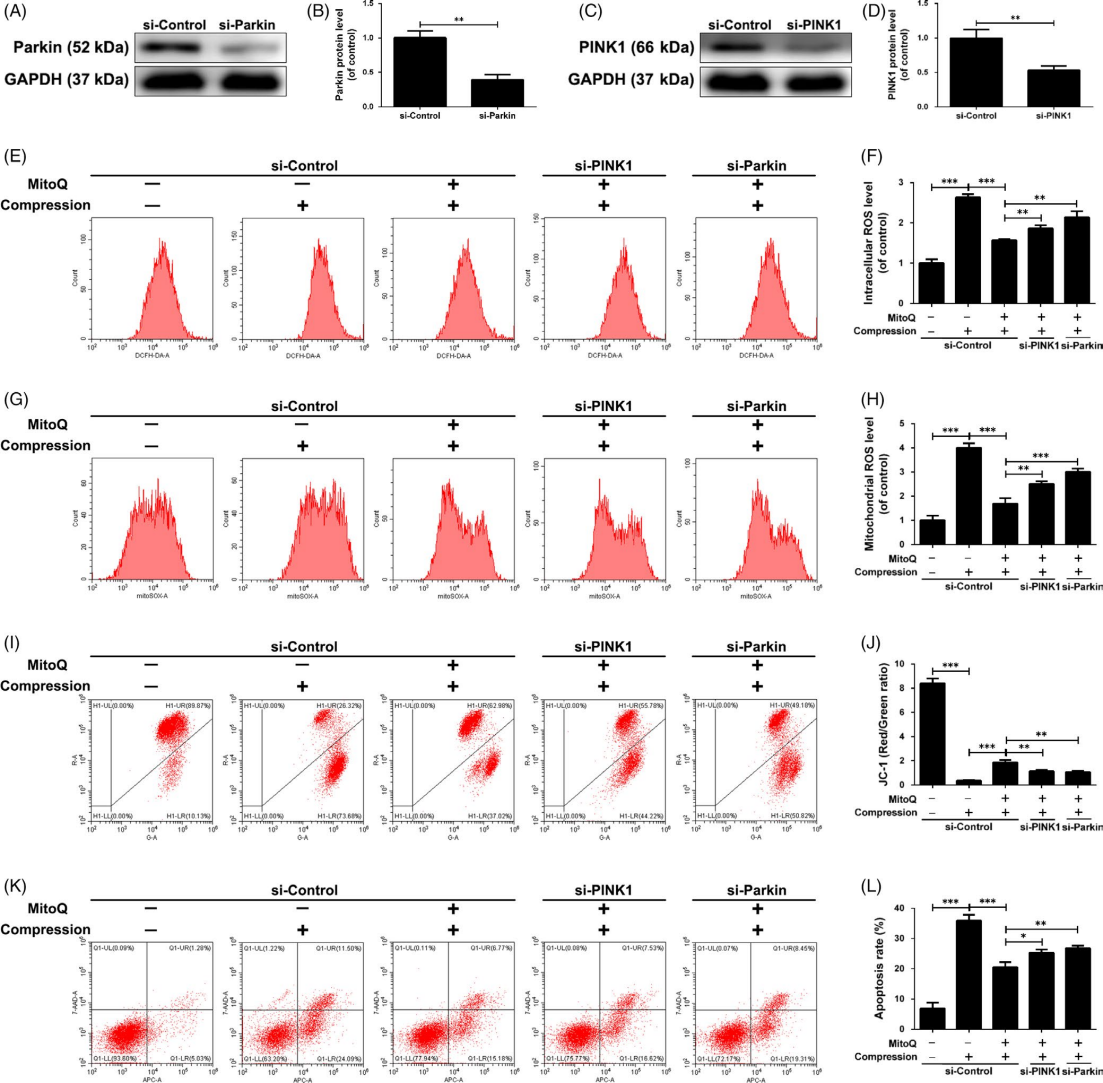
PINK1/Parkin‐mediated mitophagy is involved in the beneficial effects of MitoQ on compression‐exposed human NP cells. Human NP cells were transfected with siPINK1 (100 nmol/L) or siParkin (100 nmol/L) for 48 h followed by the administration of MitoQ and compression. (A‐B) The protein levels of Parkin in the human NP cells were measured by Western blotting. (C‐D) The protein levels of PINK1 in the human NP cells were measured by Western blotting. (E‐F) The intracellular ROS levels in the human NP cells were detected using the DCFH‐DA and measured by flow cytometry. (G‐H) The mitochondrial ROS levels were detected using the MitoSOX Red and measured by flow cytometry. (I‐J) Mitochondrial membrane potential was detected by JC‐1 staining and measured by flow cytometry. (K‐L) Annexin V‐APC/7‐AAD staining results showing the rate of apoptosis in human NP cells. Data are represented as the mean ± SD. ***P < .001, **P < .01, *P < .05, n = 3

Then, we knocked down PINK1 and Parkin using siRNAs to interfere with mitophagy activation. As shown in Figure 7A‐D, the expression of PINK1 and Parkin in human NP cells was effectively suppressed by transfection with siRNAs targeting PINK1 and Parkin, respectively. In addition, the results showed that MitoQ suppressed cellular ROS production (Figure 7E,F), mitochondrial ROS production (Figure 7G,H) and apoptosis (Figure 7K,L), whereas it increased ΔΨm (Figure 7I,J) compared with cells exposed to compression alone; however, these effects were partially abrogated by siPINK1 or siParkin transfection (Figure 7E‐L).

To investigate the potential contribution of mitophagic flux restoration to the beneficial effect of MitoQ, human NP cells were treated with the recognized autophagic flux inhibitor chloroquine (CQ), which suppresses lysosomal acidification and impairs autophagosome‐lysosome fusion. CQ compromised the protective effects of MitoQ on oxidative stress, mitochondrial dysfunction and apoptosis induced by compression in human NP cells (Figure S1A‐H). Thus, mitophagy upregulation coupled with mitophagic flux enhancement can effectively prevent the accumulation of damaged mitochondria, increase in ROS production and upregulation of apoptosis in compression‐exposed human NP cells.

### 3.7 Effects of MitoQ on the Nrf2 pathway in compression‐exposed human NP cells

**Figure 8:**
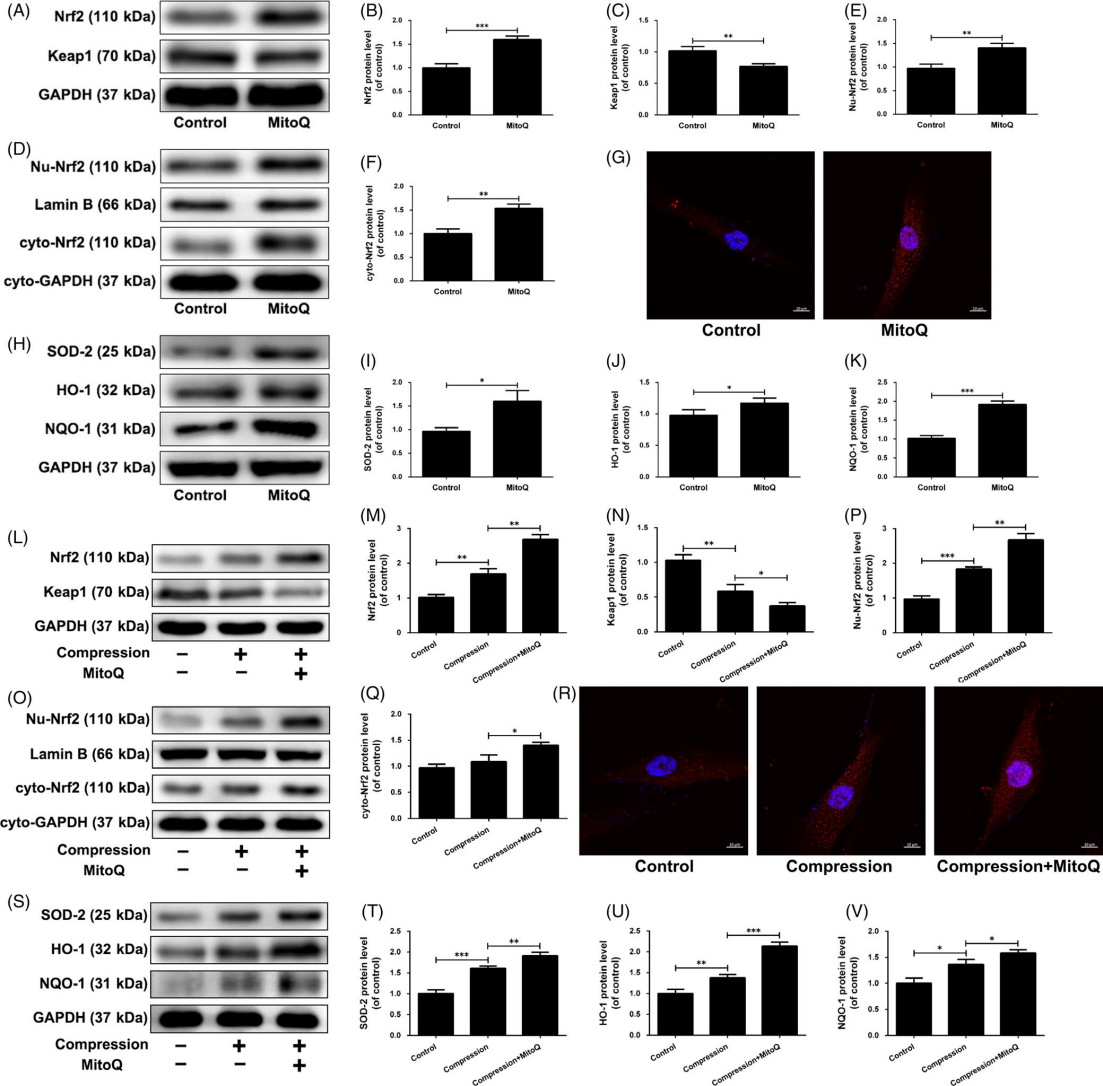
Effect of MitoQ on the Nrf2 signalling in compression‐exposed human NP cells. (A‐J) Human NP cells were treated with or without MitoQ (500 nmol/L) for 12 h. (A‐C) The protein levels of Nrf2 and Keap1 in the human NP cells were measured by Western blotting. (D‐F) The protein expression of nucleus Nrf2 (Nu‐Nrf2) and cytoplasmic Nrf2 (Cyto‐Nrf2) was measured by Western blotting. (G) The nuclear translocation of Nrf2 was examined by immunofluorescence on confocal microscope. Scale bar: 10 μm. (H‐K) The protein levels of SOD‐2, HO‐1 and NQO‐1 in the human NP cells were measured by Western blotting. (L‐V) Human NP cells were pre‐treated with MitoQ (500 nmol/L) for 2 h and then exposed to compression for 36 h. (L‐Q) The protein levels of Nrf2, Keap1, Nu‐Nrf2 and cyto‐Nrf2 in the human NP cells were measured by Western blotting. (R) The nuclear translocation of Nrf2 was examined by immunofluorescence on confocal microscope. Scale bar: 10 μm. (S‐V) The protein levels of SOD‐2, HO‐1 and NQO‐1 in the human NP cells were measured by Western blotting. Data are represented as the mean ± SD. ***P < .001, **P < .01, *P < .05, n = 3

The above results indicated that MitoQ markedly alleviated the oxidative stress induced by compression in human NP cells. Therefore, it was reasonable to assume that the effect of MitoQ would be mediated in part by classical Nrf2 anti‐oxidative signalling activation. Western blot results showed that Keap1 protein expression was downregulated and Nrf2 protein expression was upregulated in human NP cells after MitoQ treatment compared with the control group (Figure 8A‐C). Additionally, protein expression of Nrf2 in the nucleus and cytoplasm was increased in MitoQ‐treated human NP cells, as indicated by Western blotting (Figure 8D‐F) and immunofluorescence (Figure 8G). Next, since these Nrf2‐dependent anti‐oxidants, including HO‐1, SOD‐2 and NQO‐1, were selected in previous studies to determine Nrf2 activity and function in NP cells, we detected their expression levels in MitoQ‐treated cells in this study. Similar to Nrf2, HO‐1, SOD‐2 and NQO‐1 protein levels were increased in MitoQ‐treated cells compared with control NP cells (Figure 8H‐K). These results proved that MitoQ inhibited Keap1 to activate Nrf2 signalling cascade in human NP cells.

Further, we measured the activity of Nrf2 signalling in human NP cells exposed to compression and treated with MitoQ. As shown in Figure 8L‐V, compression increased the protein expression of Nrf2 and its downstream targets and the nuclear translocation of Nrf2. MitoQ treatment further increased the activity of Nrf2 pathway in compression‐treated human NP cells (Figure 8L‐V). Overall, these findings suggested that MitoQ can activate the Nrf2 signalling pathway in compression‐exposed human NP cells.

### 3.8 Role of Nrf2 signalling in MitoQ‐mediated anti‐oxidative stress and anti‐mitochondrial disorder in compression‐exposed human NP cells

**Figure 9:**
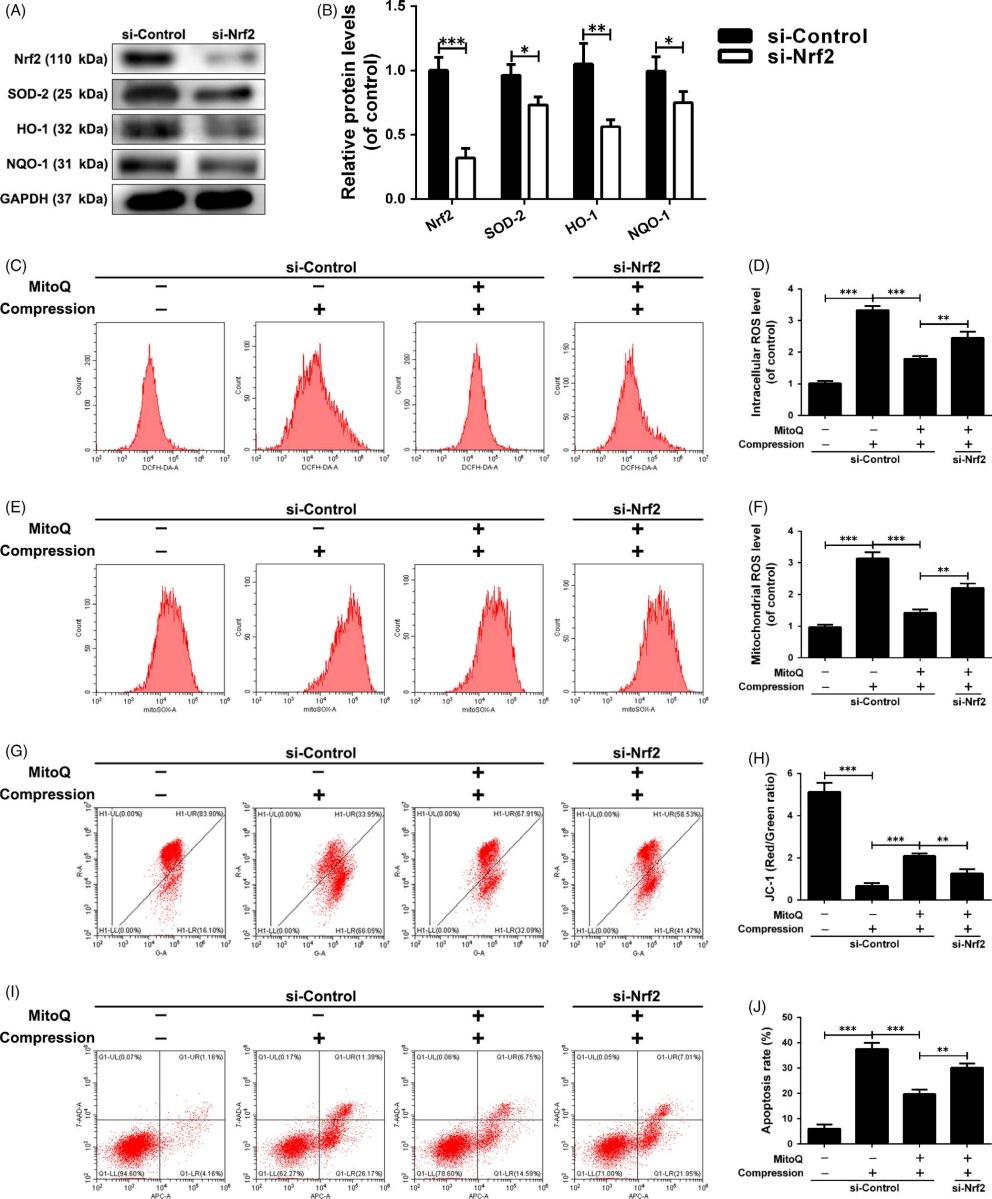
Role of Nrf2 signalling in MitoQ‐mediated anti‐oxidative stress and anti‐mitochondrial disorder in compression‐exposed human NP cells. Human NP cells were transfected with siNrf2 (100 nmol/L) for 48 h followed by the administration of MitoQ and compression. (A‐B) The protein levels of Nrf2, SOD‐2, HO‐1 and NQO‐1 in the human NP cells were measured by Western blotting. (C‐D) The intracellular ROS levels in the human NP cells were detected using the DCFH‐DA and measured by flow cytometry. (E‐F) The mitochondrial ROS levels were detected using the MitoSOX Red and measured by flow cytometry. (G‐H) Mitochondrial membrane potential was detected by JC‐1 staining and measured by flow cytometry. (I‐J) Annexin V‐APC/7‐AAD staining results showing the rate of apoptosis in human NP cells. Data are represented as the mean ± SD. ***P < .001, **P < .01, *P < .05, n = 3

To determine whether and how Nrf2 mediates the protective effects of MitoQ on human NP cells under compression, we specifically knocked down Nrf2 in human NP cells followed by MitoQ treatment and compression exposure. Western blot results showed that transfection with siNrf2 led to a significant decrease in the protein levels of Nrf2 and its downstream anti‐oxidant factors (Figure 9A,B). Nrf2 silencing partially abolished the suppressive effect of MitoQ on compression‐induced upregulation of cellular and mitochondrial ROS production and apoptotic rate and downregulation of the ΔΨm in human NP cells (Figure 9C‐J). As Nrf2 activation provides cytoprotection against oxidative stress injury by inducing several anti‐oxidant effectors and HO‐1 has been confirmed to be associated with apoptosis and extracellular matrix metabolism disorder in NP cells under pathological conditions, we directly examined the beneficial effects of MitoQ by interfering with HO‐1 expression in human NP cells. Notably, siHO‐1 transfection obviously inhibited HO‐1 protein expression (Figure S2A,B) and indeed partially reversed the protective actions of MitoQ on compression‐induced oxidative stress, mitochondrial impairment and apoptosis in human NP cells (Figure S2C‐J), as did Nrf2 silencing.

### 3.9 MitoQ ameliorates IDD development in an ex vivo model

**Figure 10:**
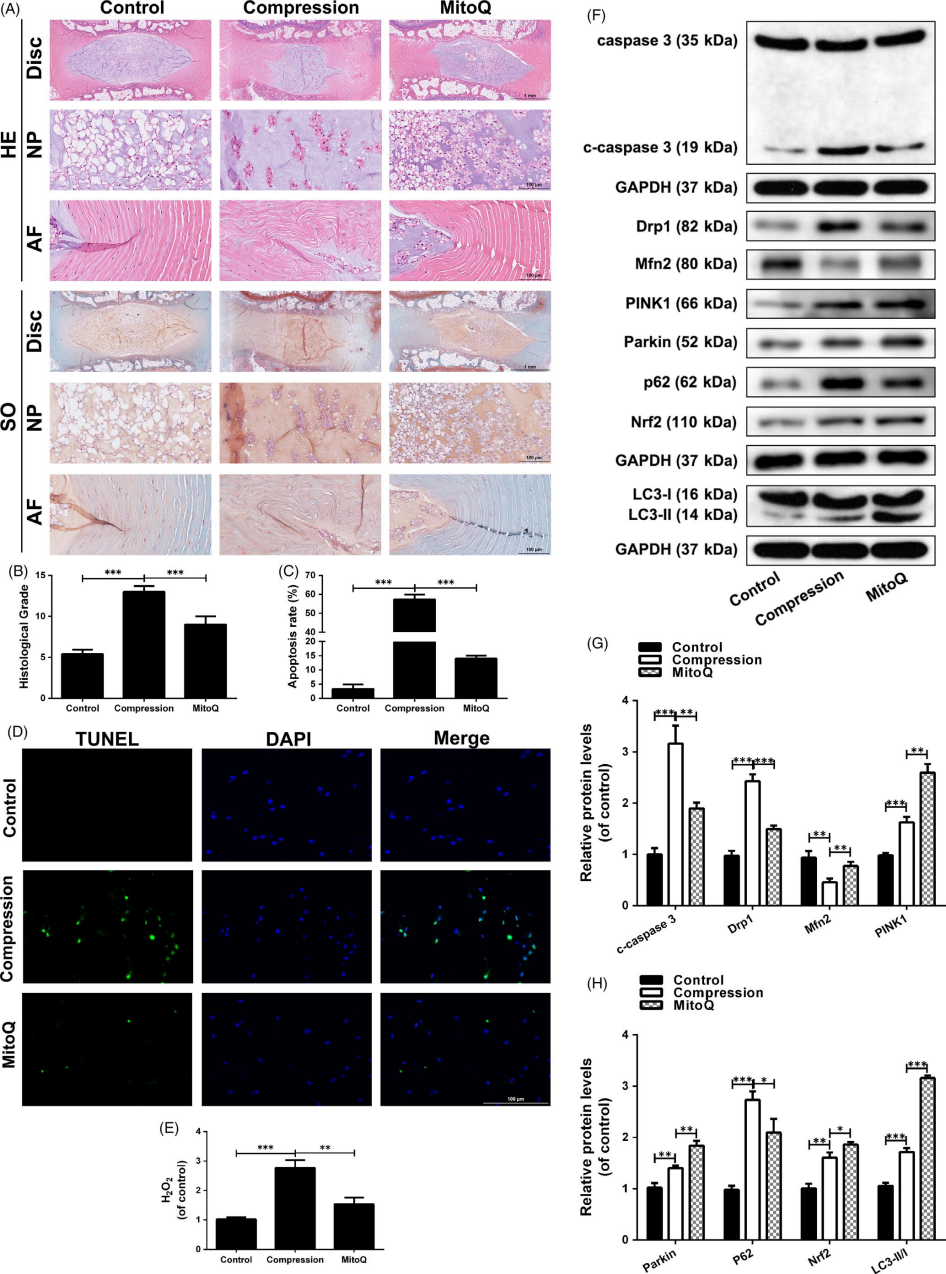
MitoQ ameliorates IDD development in an ex vivo model. The rat IVDs from compression group were cultured under compression treatment for 2 wk. The rat IVDs from MitoQ group were cultured under compression and MitoQ cotreatment for 2 wk. (A) HE and SO staining of the rat IVD tissues. (B) The histological scores of the rat IVD tissues according to histological grading scale. (C‐D) TUNEL staining and fluorescence microscope analysis were used to evaluate the apoptosis in the rat IVD tissues. Scale bar: 100 μm. (E) The content of H_2_O_2_ in the disc samples. (F‐H) The levels of caspase‐3, c‐caspase 3, Drp1, Mfn2, PINK1, Parkin, P62, Nrf2 and LC3 proteins in the rat IVD tissues were measured by Western blotting. Data are represented as the mean ± SD. ***P < .001, **P < .01, *P < .05, n = 6

To further confirm the findings of these in vitro experiments, we investigated the efficacy of MitoQ as a therapeutic agent for IDD in an ex vivo compression model of rat tail disc degeneration. After 2 weeks of compression treatment, the disc tissues were harvested and evaluated by HE and SO staining. Compared to the control group, the compression treatment group showed various degenerative changes, including a reduction in NP size, inward bulging and disorganization of the inner AF tissues and dense extracellular matrix (Figure 10A). With MitoQ treatment, these degenerative changes of the disc structure were alleviated (Figure 10A). Histological scores also indicated that MitoQ protected against IDD development (Figure 10B). The results of TUNEL staining in IVD specimens from rats revealed that the apoptosis rate was decreased in the MitoQ group compared with the compression group (Figure 10C,D). Meanwhile, compared with the compression group, there was a significant decrease in H_2_O_2_ content in the MitoQ treatment group (Figure 10E). In the MitoQ group, the levels of Mfn2, PINK1, Parkin, Nrf2 and LC3‐II/I were increased and those of cleaved caspase‐3, Drp1 and p62 were decreased when compared to the compression group as indicated by Western blotting (Figure 10F‐H). Immunohistochemical staining results for cleaved caspase‐3, Drp1, Mfn2, Parkin, LC3, p62 and Nrf2 were consistent with the Western blotting results (Figure S3). Together, these findings suggested that MitoQ ameliorates IDD progression in an ex vivo model, likely by improving mitochondrial dynamics, mitophagic flux and Nrf2 signalling*.*

## 4 DISCUSSION

In this study, we constructed the IDD model induced by compression and found that compression leads to mitochondrial dysfunction, oxidative stress and apoptosis in human NP cells. MitoQ is a mitochondria‐targeted anti‐oxidant that has therapeutic effects in numerous mitochondria‐related diseases. Therefore, we used MitoQ as a stabilizer of mitochondrial function to treat compression‐exposed human NP cells in this study. We found that MitoQ prevented ROS overproduction, mitochondrial impairment and apoptosis induced by compression in human NP cells. Next, an ex vivo compression model of IDD was used in this study. The ex vivo culture system, including the IVD and adjacent endplates, allows nutrients and outer substances to penetrate into the inner disc while maintaining the integrity of the IVD structure and native extracellular matrix.^54^, ^55^, ^56^, ^57^ Compared to in vitro study, the ex vivo study, which utilized IVD explants, offers advantages for investigating the pathological mechanisms of IDD.^54^, ^55^ Our ex vivo study showed that MitoQ treatment ameliorated the development of IDD, which confirmed the protective effect of MitoQ on NP cells in vitro. Additionally, we investigated the roles of mitochondrial dynamics, mitophagy and Nrf2 signalling in NP cell apoptosis under compression.

Under normal physiological conditions, mitochondria undergo morphological changes to meet cellular energy requirements. These changes can occur through sustained fusion and fission. Excessive mitochondrial fission leads to fragmentation of mitochondria, resulting in mitochondrial dysfunction and ultimately cell death.^58^ Sugioka et al^59^ reported that knockdown of Mfn1 or Mfn2 resulted in mitochondrial fragmentation and increased sensitivity to apoptotic stimuli. Kim et al^60^ reported that inhibition of Drp1‐mediated mitochondrial fission protected adult rat hippocampal neural stem cells against palmitate‐induced oxidative stress and apoptosis by preserving mitochondrial integrity. We found that compression not only decreased the expression of the mitochondrial fusion proteins Mfn1, Mfn2 and Opa1, but also increased the expression of Drp1 and its receptors Mff and Fis1 and the mitochondrial translocation of Drp1 in human NP cells. However, administration of MitoQ significantly restored order to mitochondrial dynamics. Furthermore, addition of a mitochondrial fission agonist and blocker revealed that cellular and mitochondrial ROS overproduction, mitochondrial dysfunction and apoptosis were partially attributed to an imbalance in mitochondrial dynamics in human NP cells. Hence, manipulation of the mitochondrial dynamics may be a promising treatment approach for IDD.

Mitophagy is essential for the clearance of damaged mitochondria and is an important component of mitochondrial quality control. The PINK1/Parkin pathway provides a mechanistic link between mitochondrial damage and autophagic clearance. Lack of PINK1 or Parkin results in the accumulation of dysfunctional mitochondria that release ROS, thereby increasing the oxidative stress that is involved in the pathogenesis of several diseases.^61^, ^62^, ^63^ We found that PINK1 and Parkin protein levels and mitochondrial Parkin translocation were increased in human NP cells upon compression, which was accompanied by increased colocalization of LC3 and Tom20, indicating mitophagy initiation. However, after deeper investigation, we found that the protein level of p62 was remarkably upregulated and lysosomal quenching of GFP fluorescence was compromised, indicating that compression impaired mitophagic flux. Damaged mitochondria are engulfed by autophagosomes to form mitophagosomes, which are fused with lysosomes to form mitolysosomes, where damaged mitochondria are degraded by hydrolases.^4^, ^64^ As such, disturbed lysosomal function or impaired mitophagosome‐lysosome fusion can alter mitophagic flux outcomes.^6^ Here, we found that mitophagic flux blockade resulted from impairments in mitophagosome‐lysosome fusion and lysosomal degradation ability in compression‐exposed human NP cells. Additionally, we found that MitoQ further promoted PINK1/Parkin‐mediated mitophagy and restored mitophagic flux. Moreover, in human NP cells exposed to compression and treated with MitoQ, knockdown of Parkin or PINK1 or CQ treatment sensitized NP cells to compression‐induced apoptosis, which was largely due to the inhibition of mitophagy initiation or obstruction of mitophagic flux. Our data thus provide evidence that activation of PINK1/Parkin‐mediated mitophagy combined with the enhancement of mitophagic flux is critical for human NP cell survival.

Mitochondrial dynamics and mitophagy serve as co‐ordinated quality‐control mechanisms for maintaining a healthy mitochondrial population. When mitochondria are damaged by pathological stimuli, they can fuse with healthy mitochondria to produce larger damaged mitochondria, which exacerbate the damage by releasing high levels of ROS.^4^ In this sense, co‐ordination of mitochondrial dynamics and mitophagy is very important. After the cell separates the damaged mitochondrial region through mitochondrial fission, there are two sets of daughter mitochondria that either have increased membrane potential (presumably high‐quality mitochondria) or have decreased membrane potential (presumably low‐quality mitochondria). Timely and effective clearance of low‐quality mitochondria via mitophagy contributes to the preservation of high‐quality mitochondria.^4^, ^65^, ^66^, ^67^, ^68^ In compression‐exposed human NP cells, mitochondrial fission is activated and mitophagic flux is blocked, which leads to the accumulation of damaged mitochondria, resulting in mitochondrial homeostatic imbalance and apoptosis. We used MitoQ to maintain the balance of mitochondrial dynamics and relieve the blockage of mitophagic flux, which reversed mitochondrial dysfunction and NP cell apoptosis.

Oxidative stress is closely associated with inadequate activation of the anti‐oxidant system.^23^, ^69^ Under resting conditions, Keap1 binds to cytosolic Nrf2, promoting continual ubiquitination and proteasomal degradation of Nrf2 through a Cul3‐ubiquitin ligase complex. Upon dissociated from Keap1, Nrf2 is translocated into the nucleus and then initiates cytoprotective gene transcription to counteract oxidative stress and modulate redox status balance.^23^ In keeping with this, the Nrf2‐activating drug dimethyl fumarate has been approved by the FDA for the treatment of multiple sclerosis,^70^ in part based on its anti‐oxidative effects. Further, Nrf2 plays an important role in maintaining mitochondrial homeostasis by affecting the ΔΨm, respiration, oxidative phosphorylation, ATP synthesis, mitochondrial biogenesis and mitochondrial integrity.^24^, ^71^, ^72^ Here, we observed increases in the activity of Nrf2 pathway in human NP cells upon compression, reflecting the NP cellular response to the induced oxidative stress. However, this increase in Nrf2 signalling activity was not sufficient to counteract the pathogenic events induced by oxidative stress in compression‐exposed human NP cells. Moreover, MitoQ further promoted Nrf2 expression and activity in human NP cells exposed to compression. siRNA‐mediated depletion of Nrf2 and HO‐1 partially inhibited the protective actions of MitoQ on compression‐induced oxidative stress, mitochondrial impairment and apoptosis. These results suggest that the beneficial effects of MitoQ on compression‐exposed human NP cells are partially attributed to the upregulation of Nrf2 expression and activity.

## 5 CONCLUSION

This study demonstrated the beneficial effects of MitoQ on human NP cell apoptosis in IDD in in vitro and ex *vivo* models. The underlying mechanism was found to be closely associated with the maintenance of mitochondrial homeostasis and redox balance through restoration of the mitochondrial fission/fusion balance and amelioration of the mitophagic flux disturbance as well as activation of Nrf2 signalling, all of which eventually promoted the survival of human NP cells (Figure S4). These results suggest that restoring mitochondrial functions and eradicating oxidative insults represent a promising therapeutic strategy for IDD and that MitoQ might serve as an effective therapeutic agent for this disorder.

## CONFLICT OF INTEREST

These authors have no conflict of interest to declare.

## AUTHOR CONTRIBUTIONS

Yuan Xue and Xiaozhi Liu conceived and designed the experiments. Liang Kang, Shiwei Liu, Jingchao Li, Yueyang Tian, Yuan Xue and Xiaozhi Liu performed the experiments. Liang Kang, Shiwei Liu, Jingchao Li and Yueyang Tian analysed the data. Liang Kang and Yuan Xue wrote the paper. Liang Kang, Shiwei Liu, Jingchao Li, Yueyang Tian, Yuan Xue and Xiaozhi Liu reviewed and revised the manuscript. All authors have read and approved the final version of the manuscript.

## Data Availability

The data that support the findings of this study are available from the corresponding author upon reasonable request.
