# Supplementary figures and images for: The mitochondria‐targeted anti‐oxidant MitoQ protects against intervertebral disc degeneration by ameliorating mitochondrial dysfunction and redox imbalance
Source: Cell Prolif. 2020 Feb 5;53(3):e12779. doi: 10.1111/cpr.12779 (PMC7106957; doi:10.1111/cpr.12779)

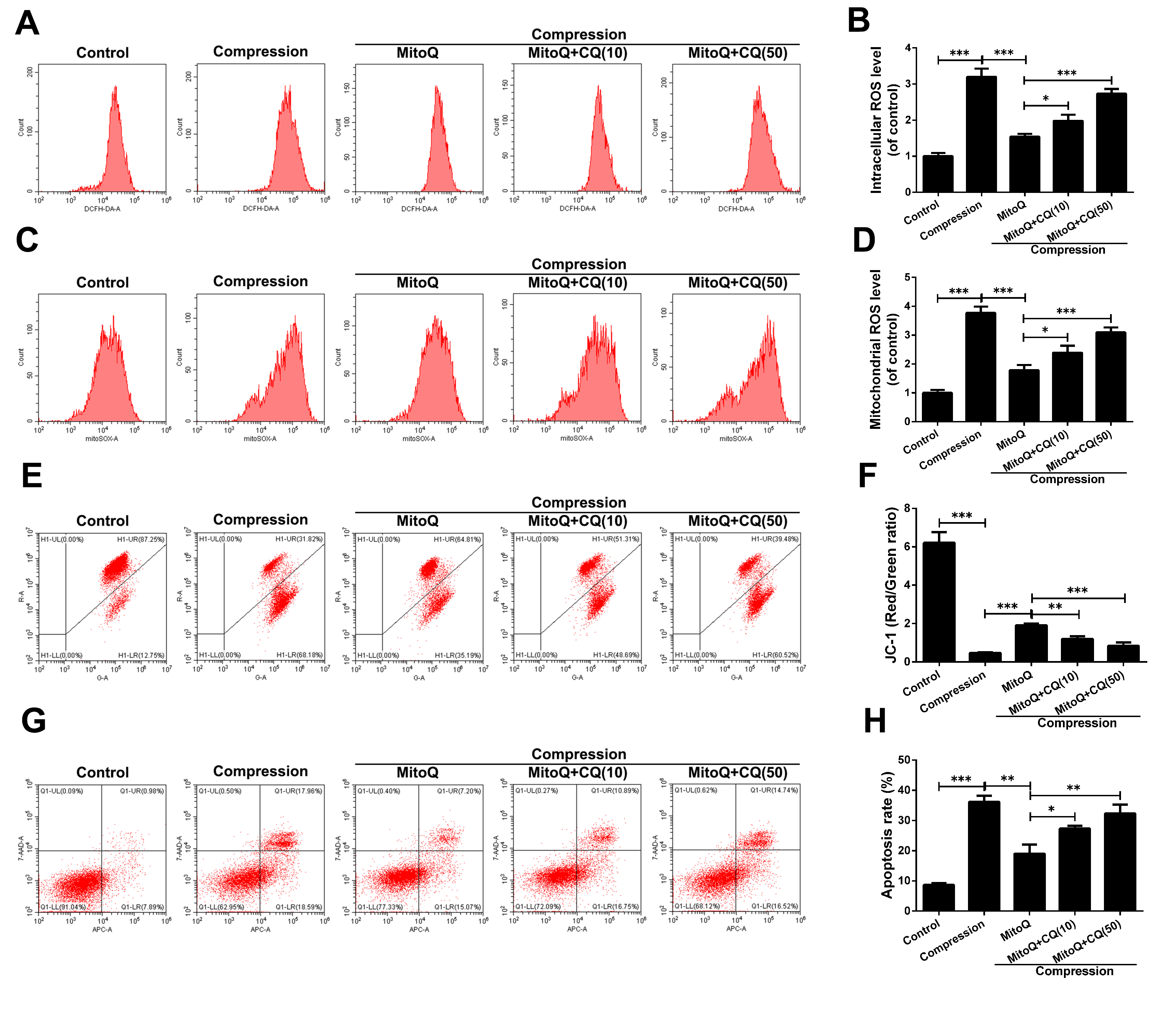

Supplement: Supplementary file 1 [file CPR-53-e12779-s001.tif]

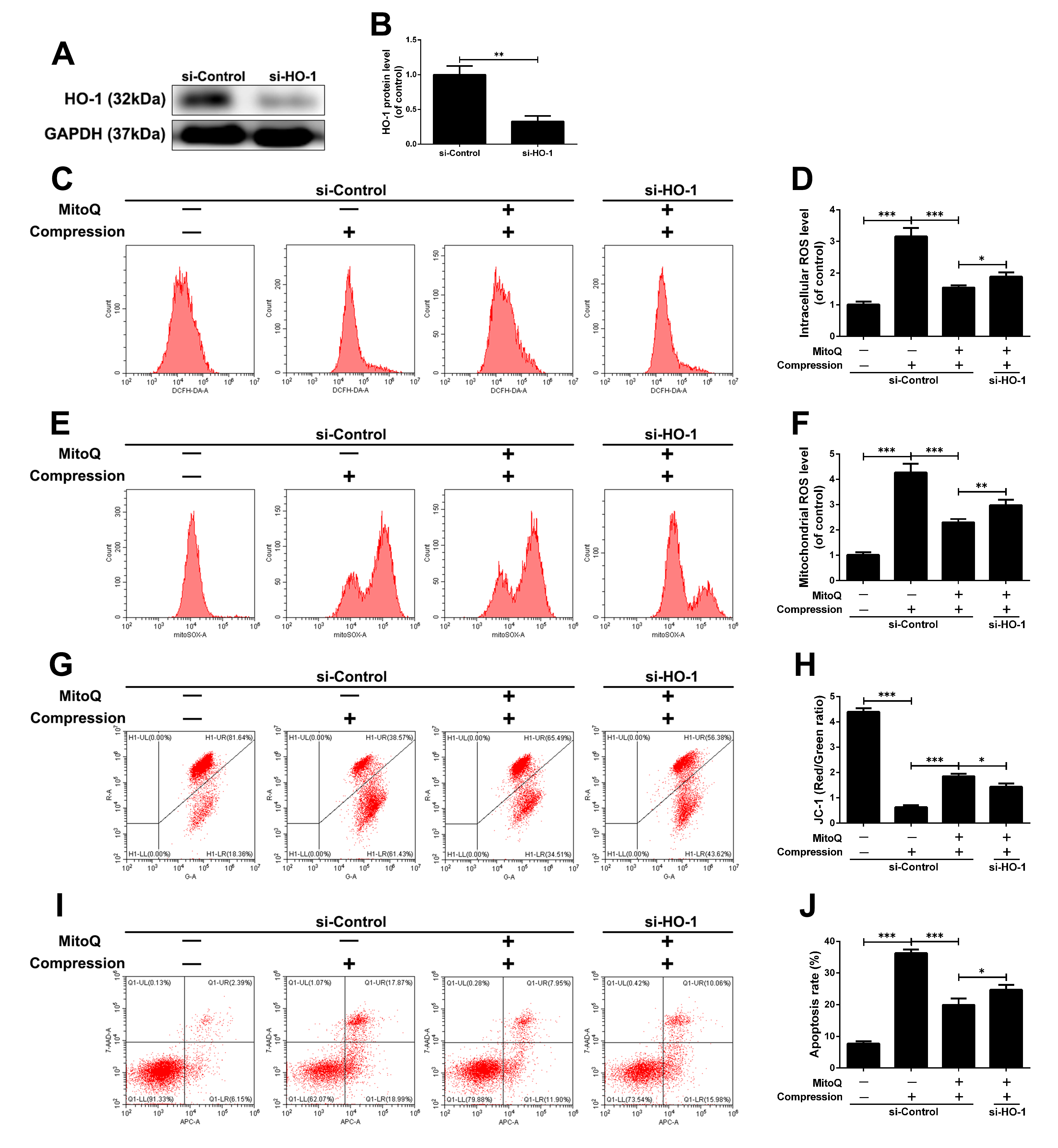

Supplement: Supplementary file 2 [file CPR-53-e12779-s002.tif]

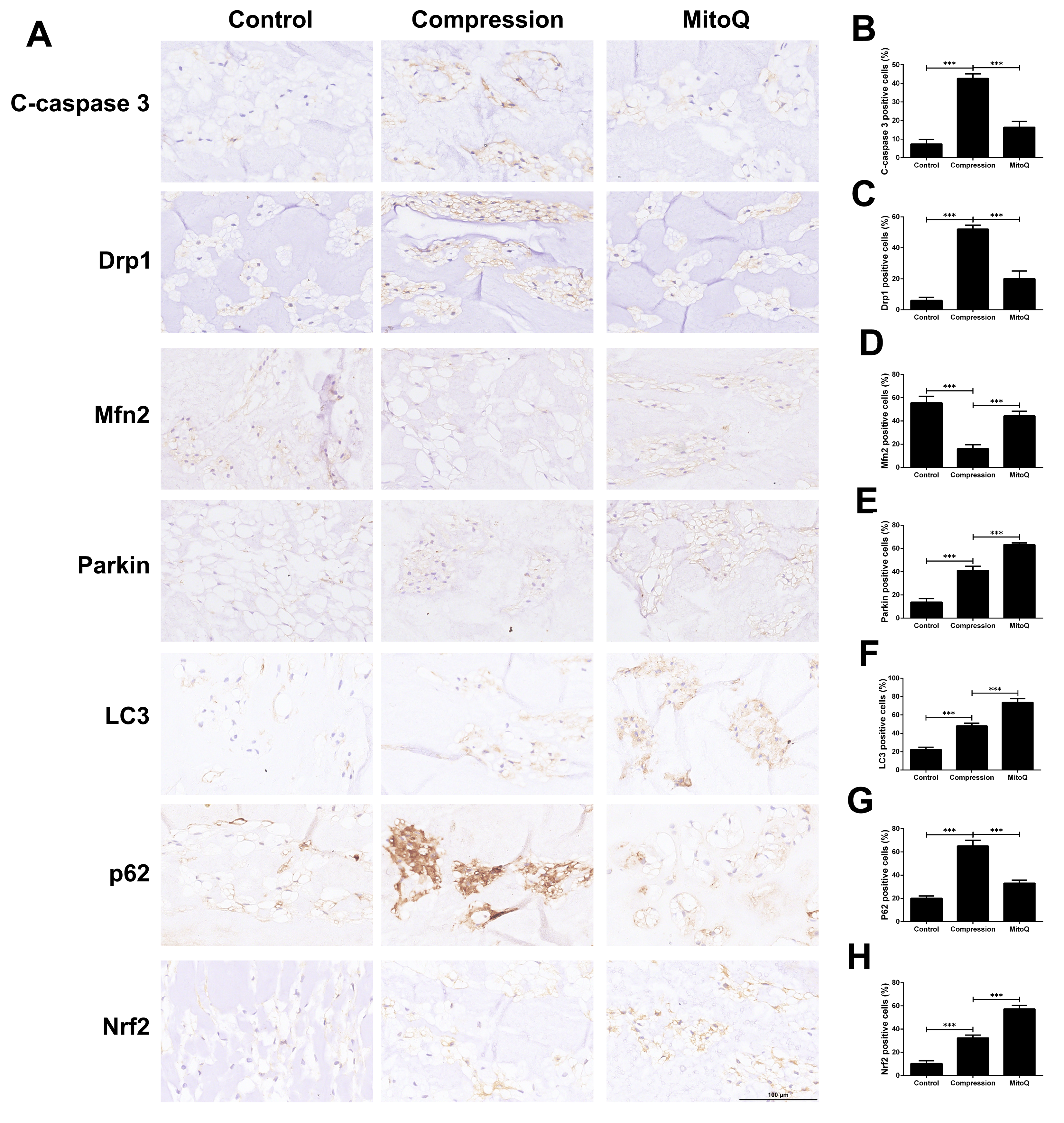

Supplement: Supplementary file 3 [file CPR-53-e12779-s003.tif]

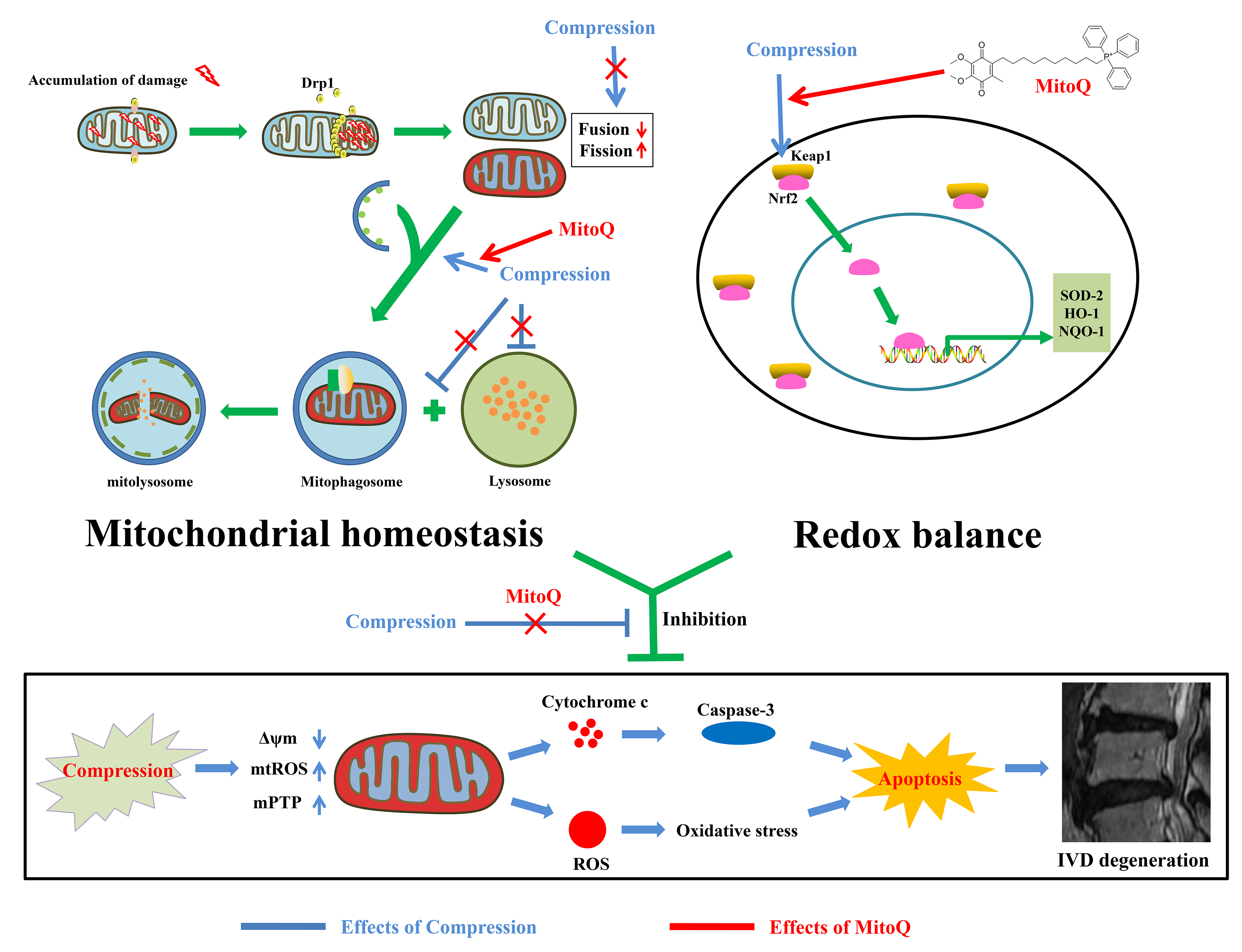

Supplement: Supplementary file 4 [file CPR-53-e12779-s004.tif]
