# Supplementary material for: The mitochondria‐targeted anti‐oxidant MitoQ protects against intervertebral disc degeneration by ameliorating mitochondrial dysfunction and redox imbalance
Source: Cell Prolif. 2020 Feb 5;53(3):e12779. doi: 10.1111/cpr.12779 (PMC7106957; doi:10.1111/cpr.12779)
Supplement: Supplementary file 5 [file CPR-53-e12779-s005.docx]

**Supplementary Figure legends**

**Figure S1.** Role of mitophagic flux restoration in the beneficial effect of MitoQ in compression-exposed human NP cells. Human NP cells were pretreated with different concentrations of CQ (10 μM, 50 μM) for 2 h followed by the administration of MitoQ and compression. (A-B) The intracellular ROS levels in the human NP cells were detected using the DCFH-DA and measured by flow cytometry. (C-D) The mitochondrial ROS levels were detected using the MitoSOX Red and measured by flow cytometry. (E-F) Mitochondrial membrane potential was detected by JC-1 staining and measured by flow cytometry. (G-H) Annexin V-APC/7-AAD staining results showing the rate of apoptosis in human NP cells. Data are represented as the mean ± SD. ***P < 0.001, **P < 0.01, *P < 0.05, n=3.

**Figure S2.** Knockdown of HO-1 compromises the beneficial effects of MitoQ on compression-exposed human NP cells. Human NP cells were transfected with siHO-1 (100 nM) for 48 h followed by the administration of MitoQ and compression. (A-B) The protein levels of HO-1 in the human NP cells were measured by western blotting. (C-D) The intracellular ROS levels in the human NP cells were detected using the DCFH-DA and measured by flow cytometry. (E-F) The mitochondrial ROS levels were detected using the MitoSOX Red and measured by flow cytometry. (G-H) Mitochondrial membrane potential was detected by JC-1 staining and measured by flow cytometry. (I-J) Annexin V-APC/7-AAD staining results showing the rate of apoptosis in human NP cells. Data are represented as the mean ± SD. ***P < 0.001, **P < 0.01, *P < 0.05, n=3.

**Figure S3.** Immunohistochemical staining of rat IVD tissues under compression and MitoQ treatment. (A) Immunohistochemical staining showing the expression of cleaved caspase-3, Drp1, Mfn2, Parkin, LC3, P62, and Nrf2 proteins in the rat IVD tissues. Scale bar: 100 μm. (B-H) Relative positive cells of cleaved caspase-3, Drp1, Mfn2, Parkin, LC3, P62, and Nrf2 were quantified by image pro plus. Data are represented as the mean ± SD. ***P < 0.001, n=6.

**Figure S4.** Schematic illustration of the possible mechanisms for the effects of MitoQ on oxidative stress, mitochondrial dysfunction and NP cell apoptosis in the IDD model induced by compression.
